# Antibacterial effect of copper sulfate against multi-drug resistant nosocomial pathogens isolated from clinical samples

**DOI:** 10.12669/pjms.35.5.336

**Published:** 2019

**Authors:** Lamia Benhalima, Sandra Amri, Mourad Bensouilah, Rachid Ouzrout

**Affiliations:** 1 Dr. Lamia Benhalima, Assistant Professor, Department of Biology, Universite 8 Mai 1945, Guelma, Algeria; 2 Dr. Sandra Amri, Assistant Professor, Department of Biology, Universite 8 Mai 1945, Guelma, Algeria; 3 Prof. Mourad Bensouilah, Department of Marine Biology, Universite Badji-MokhtarAnnaba, Algeria; 4 Prof. Rachid Ouzrout, Department of Marine Biology, Universite Badji-MokhtarAnnaba, Algeria

**Keywords:** Antibacterial activity, Copper sulfate, Nosocomial pathogens, Minimum bactericidal concentration, Minimum inhibitory concentration, Multi-drug resistance

## Abstract

**Background and Objective:**

With the emergence of antibiotic resistance and the hospital acquired infection, the interest for antimicrobial agents has recently increased again in public health. Copper is recommended as a supplementary method of increasing biological safety in the hospital environment. The objective of this study was to determine the antibacterial activity of copper sulfate salts on strains of bacterial pathogens isolated from different clinical pictures in different health establishment in Algeria.

**Methods:**

A total of 25 different bacterial isolates (16 *Enterobacteriaceae*, 5 *Staphylococci*, and 4 *Pseudomonas)* were tested for susceptibility to copper sulfate using minimum inhibitory concentration (MIC-Cu) and minimum bactericidal concentrations (MBC-Cu) determinations. All isolates were also tested for susceptibility to six antibiotics.

**Results:**

Antibiotic susceptibility studies revealed that 100% of isolates were resistant to one or more antibiotics. Fifty two percent of isolates were very susceptible to copper sulfate, with MICs ranging from 100 to 200 µg/ml. MBC-Cu = 1600 μg/ml showed the best bactericidal effect against the great majority of studied bacteria (52%). A good bactericidal activities of copper sulfate were recorded against *Proteus vulgaris* and *Staphylococcus aureus* (MBC/MIC=1). The Gram-negative bacteria isolates which were copper resistant also showed a high resistance to chloramphenicol (*r*=0.78) and Trimethoprime (*r*=0.61). Furthermore, the strains that were no-susceptible to three different antimicrobial classes (*Escherichia coli*, *Staphylococcus saprophyticus*) were not resistant to copper sulfate.

**Conclusion:**

Copper sulfate salts has significant antibacterial activity against multi-drug resistant nosocomial pathogens.

## INTRODUCTION

An alarming increase in antibiotic resistance among hospital pathogens has revived interest in alternative methods of reducing bioburden in healthcare facilities, focusing on the environment within hospitals.^1^ One alternative to be used as effective teat disinfection may be a copper. Living organisms requires copper at low concentrations as cofactors for metalloproteins and enzymes; however at high concentrations, Cu (II) induces an inhibition of growth in bacteria, and has a toxic effect on most microorganisms. The use of antimicrobial copper was accepted for the first time in 2008 by the United States Environmental Protection Agency.^2^ It has been shown that copper surfaces, or surfaces coated with this metal, have a 90 to 95% lower bacterial load, reducing the transmission of nosocomial infections.^3^ Moreover, recent findings that physiological Cu may be harnessed as a direct antimicrobial in innate immune cells.

Considering these antecedents, the aim of this study was to describe the antibiotic resistance and the susceptibility to copper sulfate salts of frequent nosocomial pathogens (*Enterobacteriaceae*, *Pseudomonas* and *Staphylococci*), isolated from various clinical samples from Algerian patients.

## METHODS

***Bacterial isolates*** included twenty five different strains isolated from patients from government hospital and the direction of the health of Guelma, Algeria. The strains were 7 isolates of *Escherichia coli*, two *Citrobacter freundii, two Klebsiella oxytoca, one Citrobacter diversus, one* *Yersinia enterocolitica, one* *Edwardsiella tarda, one* *Proteus vulgaris, one Salmonella typhimurium*, two *Pseudomonas fluorescens, two* *Pseudomonas aeruginosa, two* *Staphylococcus aureus, one Staphylococcus xylosus, one* *Staphylococcus epidermidis* and one *Staphylococcus saprophyticus*. The strains were isolated from blood cultures, wounds, faces and endotracheal sites of patients.

Antibiotic susceptibility was determined by the standard disk-diffusion method on Muller-Hinton (MH) agar plates (Oxoid, UK) using different antibiotic disks (Lab. Pvt. Mumbai, India).^4^ For *Enterobacteriaceae* and *Pseudomonas*, susceptibility tests with six antibiotics namely amoxicilline (20µg), cefotaxime (5µg), imipenem (10µg), gentamicin (30µg), chloramphenicol (30µg) and trimethoprime (5µg). *Staphylococci* strains were tested susceptibility to penicillin G (1U), gentamicin (10µg), vancomycine (30µg), erythromycine (15µg), chloramphenicol (30µg) and trimethoprime (5µg).

The determination of the MIC-Cu was done by the broth dilution method.^5^ Copper (II) sulfate pentahydrate (CuSO_4_ 5H_2_O) (Merck Millipore, Germany) were used to prepare 50 g/l stock solution. This stock solution was filter-sterilized and used for preparation of the final concentrations. By two-fold dilutions, concentration of Cu^2+^ was ranged from 12.5 to 1600 μg/ml. After standardization of the inoculums to 0.5 McFarland, 1ml of the diluted inoculums were added to 1ml of each metal concentration except for the sterility control.^6^ Subsequently, the tubes were incubated in an oven at 37°C for 24 hour. All the experiments were performed in duplicate. MIC-Cu determination was done visually. Microbial growth was considered as positive in the tubes that showed any increase in turbidity or growth at the bottom.^6^ The isolates were considered resistant if the MIC-Cu values exceeded that of the *Escherichia coli K12* ATCC 10798 (for *Enterobacteriaceae* isolates) *Pseudomonas aeruginosa* ATCC 27853 (for *Pseudomonas* isolates) and *Staphylococcus aureus* ATCC 25932 (for *Staphylococci* isolates) strains which were used as the control.^5^,^7^

To determine the minimum bactericidal concentration (MBC-Cu), a 10 µl from those tubes, which did not show any visible growth in MIC-Cu assay, was cultured on Mueller-Hinton agar (MHA; Oxoid, UK) and incubated at 37°C for 18 to 24 h. The lowest concentration of copper producing no growth was considered to be the minimum bactericidal concentration (MBC-Cu).

Statistical analysis was done using SPSS 25.0, one-way ANOVA test was conducted to identify the significant differences between the isolated bacteria. The correlations between the copper resistance and antibiotic resistance of different resistant strains are evaluated by the Pearson correlation coefficient (r) (p≤0.05).

## RESULTS

The obtained result revealed high level of multi-drug resistance among the isolates (Table-I). All the *Enterobacteriacea* isolates were completely resistant to amoxicillin and imipinem. It was observed that 13 (86.67%) *Enterobacteriaceae* were resistant to trimethoprime while nine (60%) and four (26.67%) were resistant to chloramphenicol and cefotaxime respectively. *Pseudomonas* isolates were completely (100%) resistant to amoxicillin, imipinem and trimethoprime. No *Enterobacteriaceae* and *Pseudomonas* strains were found to be resistant to gentamicin. All *Staphylococci* isolates, were resistant to penicillin, however, 4 (80%) and 2 (40%) were resistant to cefotaxime, vancomycin and erythromycin, respectively. The overall patterns of antimicrobial resistance showed that the major profiles included AMC/IPM/C/TMP which occurred in 28% (7/25) of bacterial isolates.

**Table I:** Resistance patterns of the bacteria isolates.

Resistance patterns	N° of antibiotics	N° of isolate	Resistant bacteria
P	1	1	*Staphylococcus aureus*
P/TMP	2	2	*Staphylococcus saprophyticus*, *Staphylococcus aureus*
AMC/IPM	2	1	*Escherichia coli*
AMC/IPM/TMP	3	4	*Escherichia coli*, *Yersinia enterocolitica*, *Klebsiella oxytoca*, *Salmonella* typhimurium.
AMC/IPM/TMP	3	2	*Pseudomonas fluorescens*, *Pseudomonas aeruginosa*
AMC/ IPM /C/TMP	4	7	*Escherichia coli*, *Citrobacter freundii*, *Citrobacter diversus*,*Klebsiella oxytoca*.
AMC/ CTX/IPM/TMP	4	2	*Escherichia coli*.
AMC/IPM/C/TMP	4	1	*Pseudomonas fluorescens*
AMC/ CTX/IPM/TMP	4	1	*Pseudomonas aeruginosa*
P/VA/E/TMP	4	1	*Staphylococcus epidermidis*
AMC/CTX/ IPM /C/TMP	5	2	*Edwardsiella tarda*, *Proteus vulgaris*.
P/GM/VA/E/C/TMP	6	1	*Staphylococcus xylosus*

P: Penicillin, TMP: Trimethoprime, AMC: Amoxicilin, IPM: Imipinem, C: Chloramphenicol,VA: Vancomycin, E: Erythromycine, CTX: Cefotaxime, GM: Gentamicin

**Fig.1:**
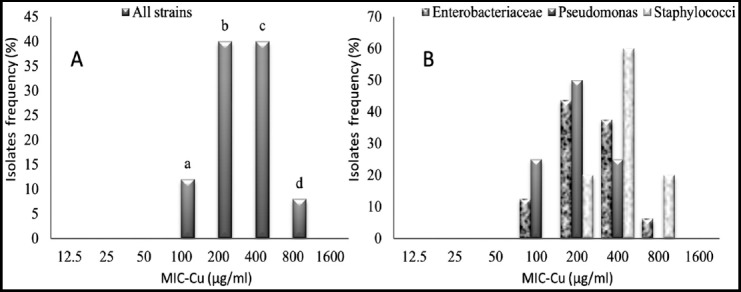
Minimum inhibitory concentration of copper (MIC-Cu) value distribution for the most prevalent isolates from Algerian patients.^a, b, c, d^ Different letters indicate no significant differences (p≤0.05) between *Enterobacteriaceae*, *Pseudomonas* and *Staphylococci* isolates under the same copper concentration according to one-way ANOVA test.

The forty percent of isolates (10/25) were inhibited by 200 μg/ml of copper sulfate. Also, the concentration 400 μg/ml of copper sulfate could inhibit 40% of isolates (10/25); however, there were two isolates (8%) that required 800 μg/ml of copper (E12: *Klebsiella oxytoca*, S4: *Staphylococcus aureus*). No growth was observed for any bacteria at 1600 μg/ml of copper (Fig. 1A). Comparison between *Enterobacteriaceae*, *Pseudomonas* and *Staphylococci* isolates to the same concentration of copper showed that the p-values were not significantly different (p>0.05).

**Fig.2:**
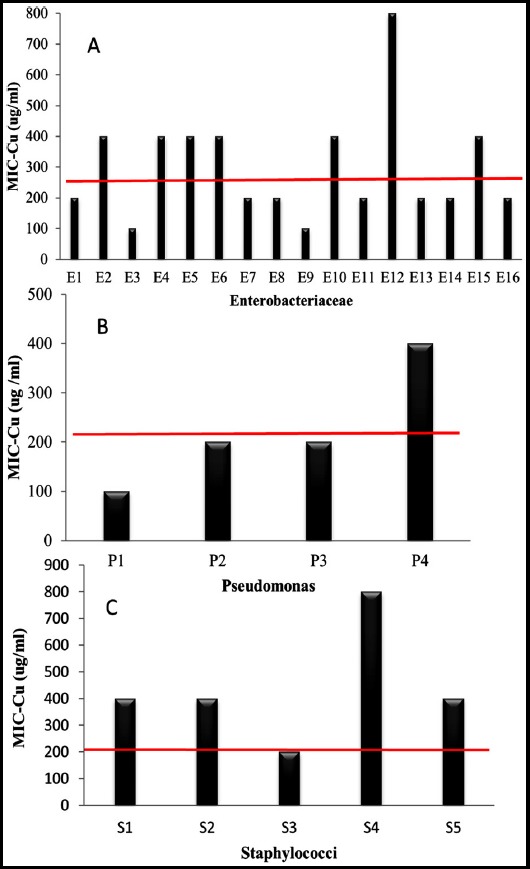
Minimum Inhibitory Concentrations of copper against nosocomial pathogenes. Horizontal line represents MIC-Cu of reference strains. E1-E7: Escherichia coli, E8-E9: *Citrobacter freundii*, E10: *Citrobacter diversus*, E11: *Yersinia enterocolitica*, E12-E13: *Klebsiella oxytoca*, E14: *Edwardsiella tarda*, E15: *Proteus vulgaris*, E16: *Salmonella Typhimurium*, P1-P2: *Pseudomonas fluorescens*, P3-P4: *Pseudomonas aeruginosa*, S1: *Staphylococcus xylosus*, S2: *Staphylococcus epidermidis*, S3: *Staphylococcus saprophyticus*, S4-S5: *Staphylococcus aureus*.

The results for *Enterobacteriaceae* isolates showed that 200 μg/ml and 400 μg/ml of copper inhibited 43.75% (7/16) and 37.5% (6/16) respectively (Fig. 1B). *Staphylococci* isolates showed slightly high MIC-Cu values than those observed for *Pseudomonas* isolates, 400 μg/ml of copper inhibited 60% (3/5) and 25% (1/4) of isolates of *Staphylococci* and *Pseudomonas*, respectively. Comparing MICs-Cu of studied bacteria and that of the standard strains, resistance for copper was found among 43.75% of the studied *Enterobacteriaceae* (Fig. 2A), one isolate of *Pseudomonas aeruginosa* (P4) (Fig. 2B) and 80% of *Staphylococci* strains (Fig. 2C).

The MBC-Cu equal to 1600 μg/ml of copper showed the best bactericidal effect against studied bacteria (52%: 13/25) (Table-II). However, a concentration of 800 μg/ml of copper was sufficient to kill 36% (9/25) of isolated strains. MBC-Cu against *Proteus vulgaris* (E15) and *Staphylococcus aureus* (S5) were found to be 400 μg/ml. As shown in Table-III, the copper sulfate salts demonstrated bactericidal or bacteriostatic effects.

**Table II:** Minimum bactericidal concentrations of copper sulfate against an important nosocomial pathogens.

	Bacterial species	MBC-Cu (µg/ml) * mean ± standard error
Gram (-)	*Escherichia coli* K12 ATCC 10798	400 ±00^a^
	*Escherichia coli*	1286±560^b^
	*Citrobacter freundii*	1200±566^c^
	*Citrobacter diversus*	1600±00^d^
	*Klebsiella oxytoca*	1600±566^e^
	*Yersinia enterocolitica*	1600±00^f^
	*Edwardsiella tarda*	800±00^g^
	*Proteus vulgaris*	400±00^h^
	*Salmonella* Typhimurium	1600±00^i^
	*Pseudomonas aeruginosa* ATCC 27853	1600±00^j^
	*Pseudomonas fluorescens*	800±00^k^
	*Pseudomonas aeruginosa*	1600±00^l^
Gram (+)	*Staphylococcus aureus* ATCC 25932	1600±00^m^
	*Staphylococcus xylosus*	800±00^n^
	*Staphylococcus epidermidis*	800±00°
	*Staphylococcus saprophyticus*	1600±00^p^
	*Staphylococcus aureus*	600±283^q^

Values followed by different letters in a column are not significantly different (p>0.05) according to ANOVA tests.

* Significant difference between Gram-positives and Gram-negatives bacteria after one-way ANOVA at a significant level of p ≤ 0.05.

**Table III:** Report MBC/MIC.

	Strains	Copper MBC/MIC	Antibacterial activity
Gram (-)	*Escherichia coli* K12 ATCC 10798	2±00	bactericidal
	*Escherichia coli*	5.6±5.1	bacteriostatic
	*Citrobacter freundii*	10±8.4	bacteriostatic
	*Klebsiella oxytoca*	4.5±4.9	bacteriostatic
	*Citrobacter diversus*	4±00	bacteriostatic
	*Yersinia enterocolitica*	8±00	bacteriostatic
	*Edwardsiella tarda*	4±00	bacteriostatic
	*Proteus vulgaris*	1±00	bactericidal
	*Salmonella* Typhimurium	8±00	bacteriostatic
	*Pseudomonas aeruginosa* ATCC 27853	8±00	bacteriostatic
	*Pseudomonas fluorescens*	6±2.8	bacteriostatic
	*Pseudomonas aeruginosa*	6±2.8	bacteriostatic
Gram (+)	*Staphylococcus aureus* ATCC 25932	8±00	bacteriostatic
	*Staphylococcus xylosus*	2±00	bactericidal
	*Staphylococcus epidermidis*	2±00	bactericidal
	*Staphylococcus saprophyticus*	8±00	bacteriostatic
	*Staphylococcus aureus*	1±00	bactericidal

## DISCUSSION

Multi-drug resistant bacteria are serious threat in clinical health settings and very challenging to treat infectious disease.^8^ From current study it was found that 84% of clinical isolates have multi-drug resistance pattern. The antibiotic resistance patterns gave interesting information indicating a selective multi-drug resistant on the clinical isolates from Algerian patients, reflects indiscriminate use of antibiotics in human medicine.

All Gram-negatives bacteria in our research found to be resistant to imipinem which is an important antibiotic for the treatment of infections caused by Gram-negatives bacteria. From recent study, it was found that carbapenem have lost their activity against *Enterobacteriaceae* and *Pseudomonas* because of two main mechanisms, carbapenemase activity and loss of porin function.^9^ Resistance of studied isolates to the chloramphenicol is unacceptably high, particularly for *Pseudomonas* isolates (100%). Chloramphenicol is an antibiotic that is rarely used due to its well-described toxicity profile. It is conceivable that high resistance rates might have been driven by overuse of this antibiotic in the study health establishment. Many studies in *Pseudomonas* confirmed the role of the efflux pump in tolerance to chloramphenicol.^10^ This study demonstrated the resistance of *Staphylococcus xylosus* and *Staphylococcus epidermidis* to vancomycin, however, in another study, vancomycin-resistant *Staphylococci* isolates were also identified in methicillin-susceptible *S. aureus*.^11^

The use of copper as an alternative to prevent nosocomial infection appears as a novel and promising idea. The biocidal effect of copper as a contact surface has been extensively investigated in a wide variety of laboratory studies and appears to have a potential application in healthcare infection prevention and control efforts. In addition to its use as a contact surface, the antimicrobial effect of copper is being exploited in a number of other settings (salts as an antibacterial particle agent). In this study, evaluation *in vitro* of the antimicrobial effectiveness of copper sulfate salts to inactivate an important nosocomial pathogens showed that a concentration as low as 800 μg/ml of copper inhibited bacterial growth in 80% of the isolates from various biological fluids from Algerian patients, including bacteria with a wide pattern of antibiotic resistance. In addition, a higher copper concentration of 1600 μg/ml should ensure inactivation, meaning it prevents the multiplication of all the isolates. The antibacterial mechanisms of copper are still being studied, but it is known to produce: inactivation of enzymatic pathways, formation of reactive oxygen species, precipitation of bacterial proteins, modification of their cell wall and destruction or alteration of the synthesis of nucleic acids, without being mutagenic.^12^ An American studies reported the activity of Cu against Gram-positive cocci such as meticillin-resistant *Staphylococcus aureus* (MRSA) and Gram-negative bacilli causing diseases, such as *Escherichia coli* O157.^13^,^14^

MBCs-Cu against Gram positive bacteria were lower than Gram-negative isolates. No significant differences between different bacterial strains were obtained from the one-way ANOVA analysis. On the contrary, significant difference was detected between Gram-positive and Gram-negative bacteria (One-way ANOVA, p<0.05). The reason for the difference in sensitivity between Gram-positive and Gram-negative bacteria might be ascribed to the differences in morphological constitutions between these microorganisms.^15^ The best bactericidal activity of copper sulfate was observed against *Proteus vulgaris, Staphylococcus aureus, Staphylococcus xylosus and Staphylococcus epidermidis*. The other strains tested recorded bacteriostatic activity. These results could be explained by the differences between strains tested.

All other compounds of copper (copper oxide, copper acetate and copper nitrate) demonstrated bacteriostatic or bactericidal properties (results not shown), but the greatest antimicrobial effectiveness for all bacteria tested was observed with copper sulfate. From the clinical point of view, the use of copper sulfate (salts or impregnated in textile or liquids) with confirmed bacteriocidal or bacteriostatic properties against multi-drug resistant nosocomial pathogens as an antibacterial agent should be very important, because there is a problem of infections and epidemics caused by these bacteria in Algerian hospitals.

One concern in use of copper sulfate as a bactericidal agent often voiced is the potential of development of resistance as has happened with antibiotics. This study could clearly demonstrate that 80% *Staphylococci*, 43.75% *Enterobacteriaceae* and one *Pseudomonas aeruginosa* strains are considered resistant to copper. This study supports previous studies suggesting that most Gram negative bacteria and *S. aureus* also encode a multi-copper oxidase, which is required for the periplasmic oxidation of Cu(I) to Cu(II).^16^,^17^

In terms of the potential for cross-resistance between copper and clinical antibiotics, the present study showed that there is good correlation among the proportion of copper-resistant Gram-negative bacteria and chloramphenicol resistance and trimethoprime resistance, with r=0.78 and r=0.61, respectively. The correlation of copper-resistant Gram-positive bacteria with penicillin resistance and trimethoprime resistance showed lower values (r=0.0123) (Table-IV). Correlation between copper resistance and resistance to chloramphenicol, trimethoprime and beta-lactams was related to four main strategies:

**Table IV:** Matrix of correlation between copper resistance and antibiotic resistance (r > 0.5 appear in bold type).

	*Gram-negative bacteria					**Gram-positive bacteria			

Resistance to	Cu	AMC	IPM	C	TMP	Resistance to	Cu	P	TMP
Cu	1					Cu	1		
AMC	0.142	1				P	0.0123	1	
IPM	0.142	**0.812**	1			TMP	0.0123	**0.9121**	1
C	**0.781**	0.012	0.012	1					
TMP	**0.618**	0.0245	0.0245	**0.6056**	1				

Cu: Copper, AMC: Ampicillin, IPM: Imipinem, C: Chloramphenicol, P: Penicillin, TMP: Trimethoprime,

* *Enterobacteriaceae* and *Pseudomonas* strains

** *Staphylococci* strains

1. Reduction of membrane permeability
2. Rapid efflux of the metal and antibiotic
3. Alteration of cellular target
4. Drug and metal sequestration.^18^

Caille *et al*. suggested that the presence of copper could increase resistance to imipenem in *P. aeruginosa* because of possible coregulation.^19^ More recently, resistance gene to copper linked to resistance genes to beta-lactam, chloramphenicol, tetracycline, fluoroquinolones and vancomycin have been found.^20^,^21^

## CONCLUSION

The copper sulfate salts has significant antibacterial activity against multi-drug resistant nosocomial pathogens. The study confirmed the effective activity (bacteriocidal or bacteriostatic) of the copper sulfate salts. This study has suggested that there was probably some correlation between the phenotype of antibiotic resistance and copper resistance.

### Author`s Contribution

**LB:** Conceived & design, statistical analysis and editing of manuscript.

**LB, SA:** Data collection and manuscript writing.

**MB, LB:** Conceptualization, methodology and writing revisions.

**RO:** Review and final approval of manuscript.
